# Pembrolizumab induced pericardial tamponade: A case report

**DOI:** 10.1002/ccr3.7298

**Published:** 2023-05-01

**Authors:** Dariusz Uczkowski, Hamza Ashraf, Arunabh Sekhri, Arbaz Samad

**Affiliations:** ^1^ Overlook Medical Center Atlantic Health System Summit New Jersey USA; ^2^ Morristown Medical Center Atlantic Health System Morristown New Jersey USA

**Keywords:** immunotherapy, lung adenocarcinoma, pembrolizumab, pericardial tamponade

## Abstract

**Key Clinical Message:**

The occurrence of a large pericardial effusion is not a commonly noted adverse event associated with pembrolizumab and our report demonstrates that a rapid development can be diagnosed with close monitoring and triage to acute medical settings.

**Abstract:**

Pembrolizumab is an immune checkpoint inhibitor used in various types of cancers. Pericardial tamponade is a rare side effect reported in only very few case reports. Early recognition and therapeutic intervention is vital in all cases. We report a case of a 54‐year‐old male with Stage 3 lung adenocarcinoma who developed cardiac tamponade secondary to pembrolizumab and subsequently required pericardial window.

## 1 INTRODUCTION

Pembrolizumab is an immunotherapy agent used in the management and treatment of various cancers. More specifically, it is a monoclonal antibody targeted against programmed death receptor‐1 (PD‐1) on lymphocytes.^1^, ^2^ PD‐1 functions as an “immune checkpoint” through protection against immune system overactivity via promotion of cellular apoptosis in antigen‐specific T cells, while also promoting the inhibition of apoptosis in regulatory T cells.^3^ Programmed death receptor ligand (PD‐L1), which is highly expressed on certain tumors cells, engages with PD‐1, and dampens the overall functional T‐cell response.^1^, ^3^ Pembrolizumab blocks the formation of the PD‐1:PDL‐1 complex, thereby preventing certain cancer cells from evading anti‐tumor immunity.^1^, ^2^ According to the Food and Drug Administration (FDA), the most common adverse reaction in patients taking pembrolizumab are fatigue, pruritis, diarrhea, decreased appetite, rash, dyspnea, constipation, and nausea, with additional warnings and precautions listed for immune‐mediated pneumonitis, colitis, hepatitis, endocrinopathies, and nephritis.^4^ The development of an effusion in the pericardial cavity, especially one large enough to invoke tamponade physiology, is an exceedingly rare adverse event of pembrolizumab and is scarcely documented in literature. We present the following case of pericardial effusion leading to cardiac tamponade requiring 600 cc drainage via pericardial window in a patient with lung adenocarcinoma treated with pembrolizumab.

## 2 CASE PRESENTATION

**FIGURE 1:**
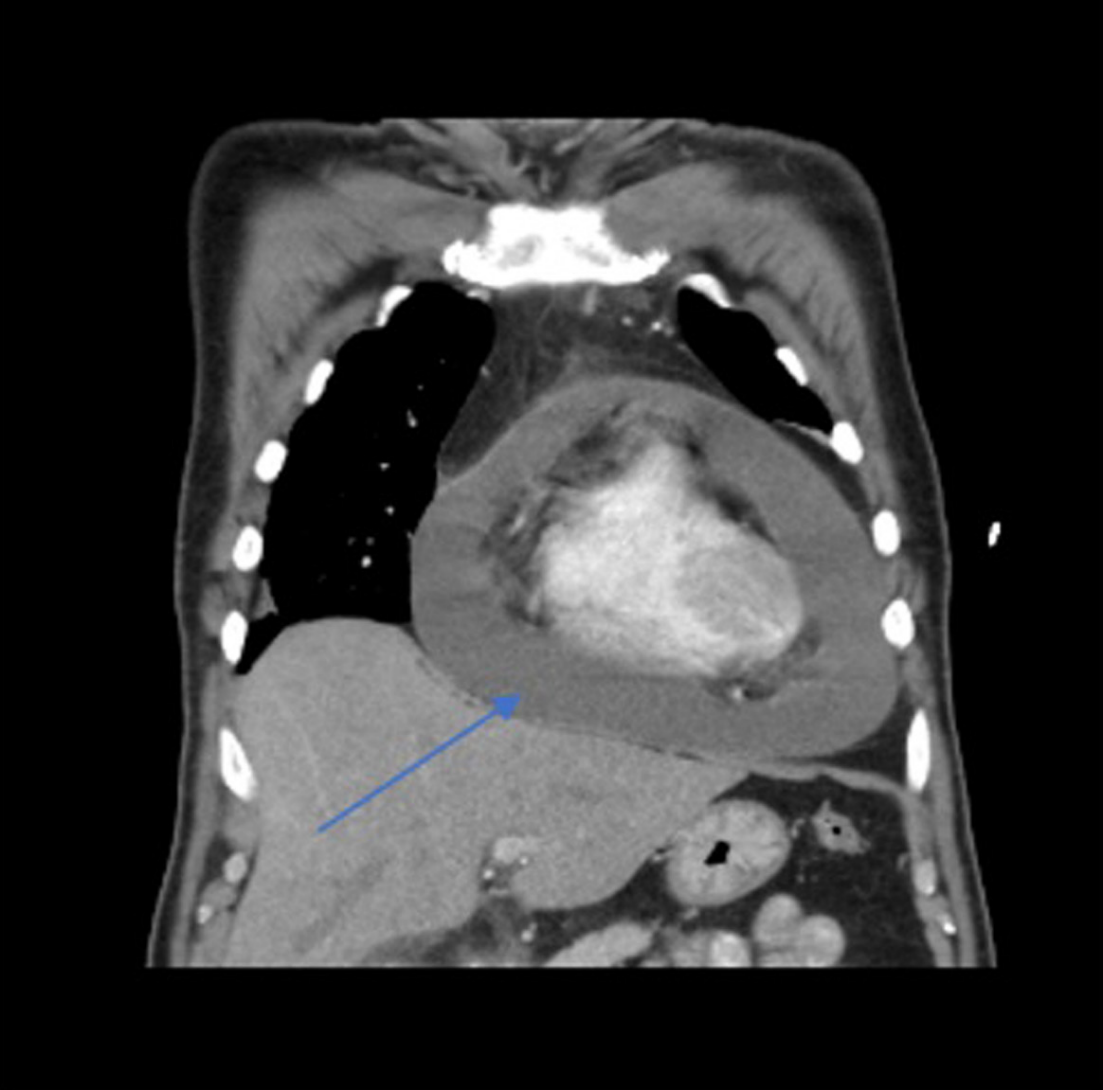
Coronal view: CT scan of the chest showing large pericardial effusion (blue arrow).

**FIGURE 2:**
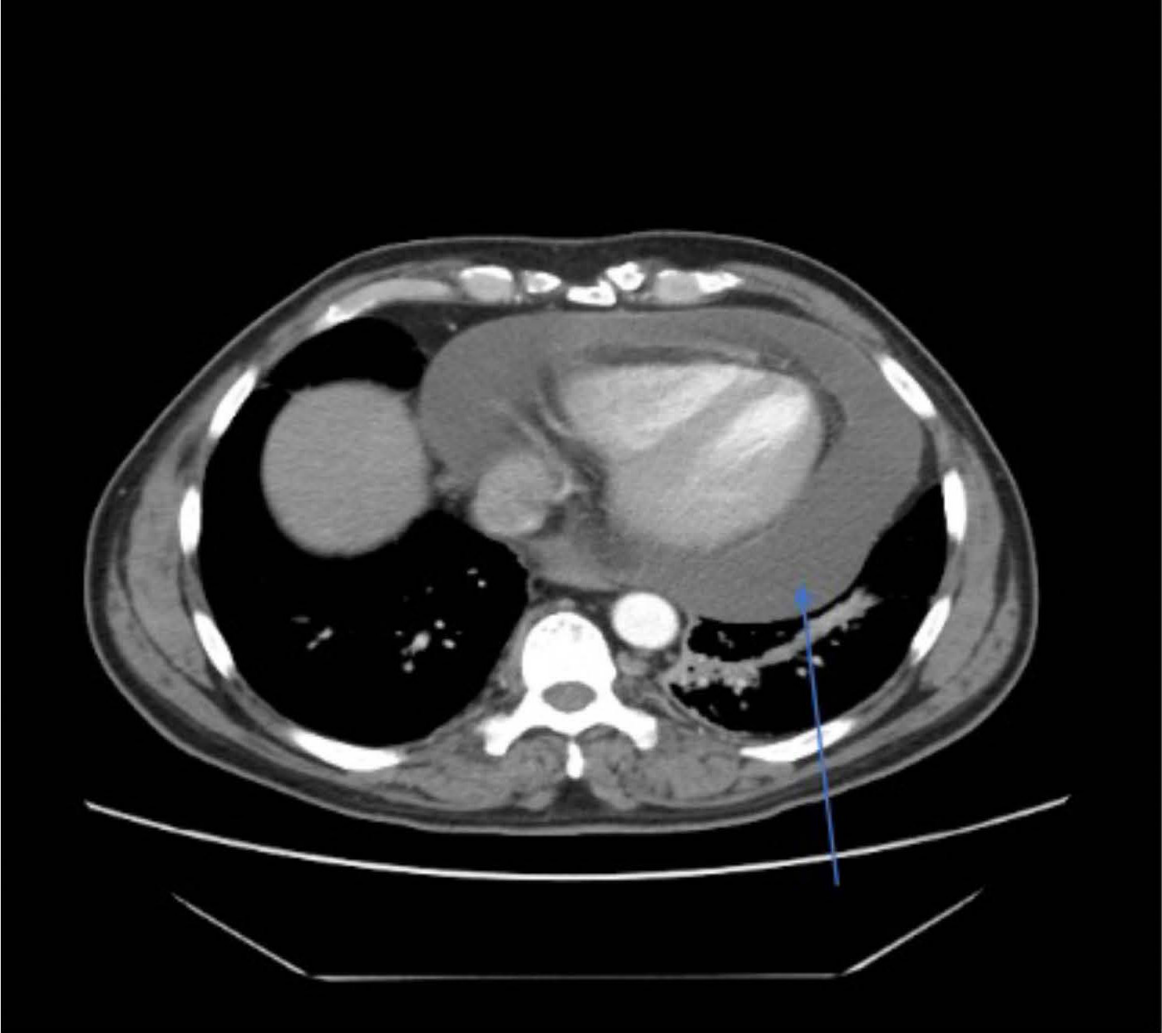
Axial plane: CT scan of chest showing large pericardial effusion (blue arrow).

A 54‐year‐old male with unresectable Stage 3 non‐small cell lung cancer (NSCLC) adenocarcinoma (T3N3) of the right upper lobe of the lung (PD‐L1 30% positive), diagnosed 3 months prior, started on carboplatin, pemetrexed, and pembrolizumab therapy with the third cycle completed 17 days prior to admission, presented to the emergency department (ED). His visit was prompted by a critical finding found on computerized tomography (CT) scan of the chest performed for monitoring of disease progression as an outpatient and for evaluation of 3 days worsening dyspnea. The patient was ultimately instructed to present to the ED by his oncologist after the imaging revealed a large pericardial effusion (Figures 1 and 2).

**FIGURE 3:**
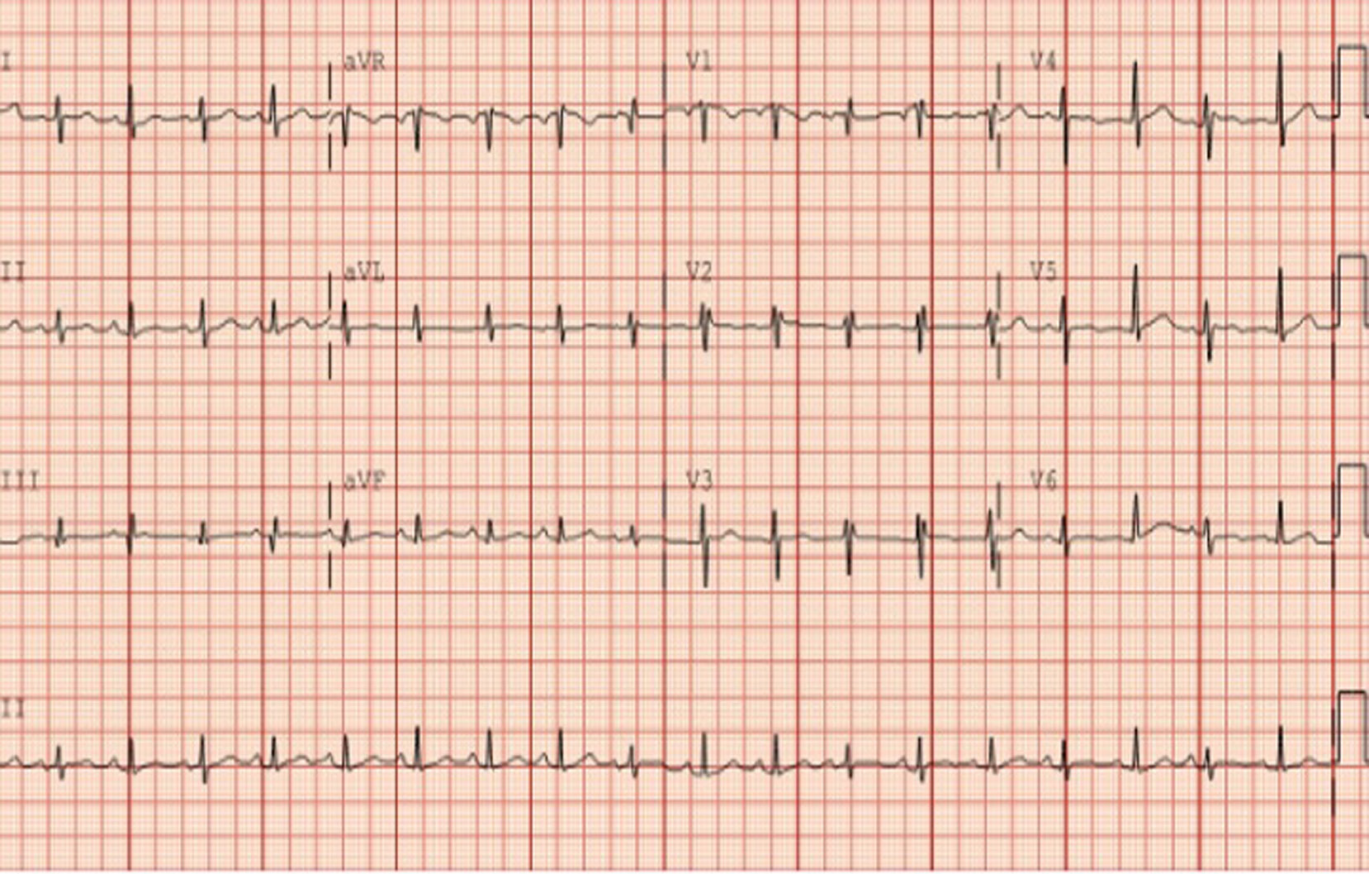
Twelve lead EKG showing sinus tachycardia at a rate of 111 beats per minute, and diffuse low‐voltage QRS complexes. EKG, electrocardiogram.

On arrival, vital signs revealed a temperature of 97.4°F, a heart rate of 89 beats per minute, a respiratory rate of 24 breaths per minute, a blood pressure of 128/38 mmHg, and an oxygen saturation of 100% on 2 L nasal cannula. An electrocardiogram revealed sinus tachycardia with low voltage QRS intervals throughout (Figure 3). Physical examination was notable for dry mucous membranes, tachycardia, muffled heart sounds and bulging neck veins. Labs on admission included a white blood cell count of 11.61 cells/nL, a hemoglobin 8.5 g/dL, a platelet count of 454 platelets/nL, sodium level of 138 mmol/L, potassium level of 4.2 mmol/L, creatinine of 1.0 mg/dL, prothrombin time 16.8 s, international normalized ratio of 1.36 s, N‐terminal pro‐brain natriuretic peptide level of 1068 pg/mL, and a thyroid‐stimulating hormone level of 0.941 ulU/mL. The patient was also found to be COVID‐19 negative.

**FIGURE 4:**
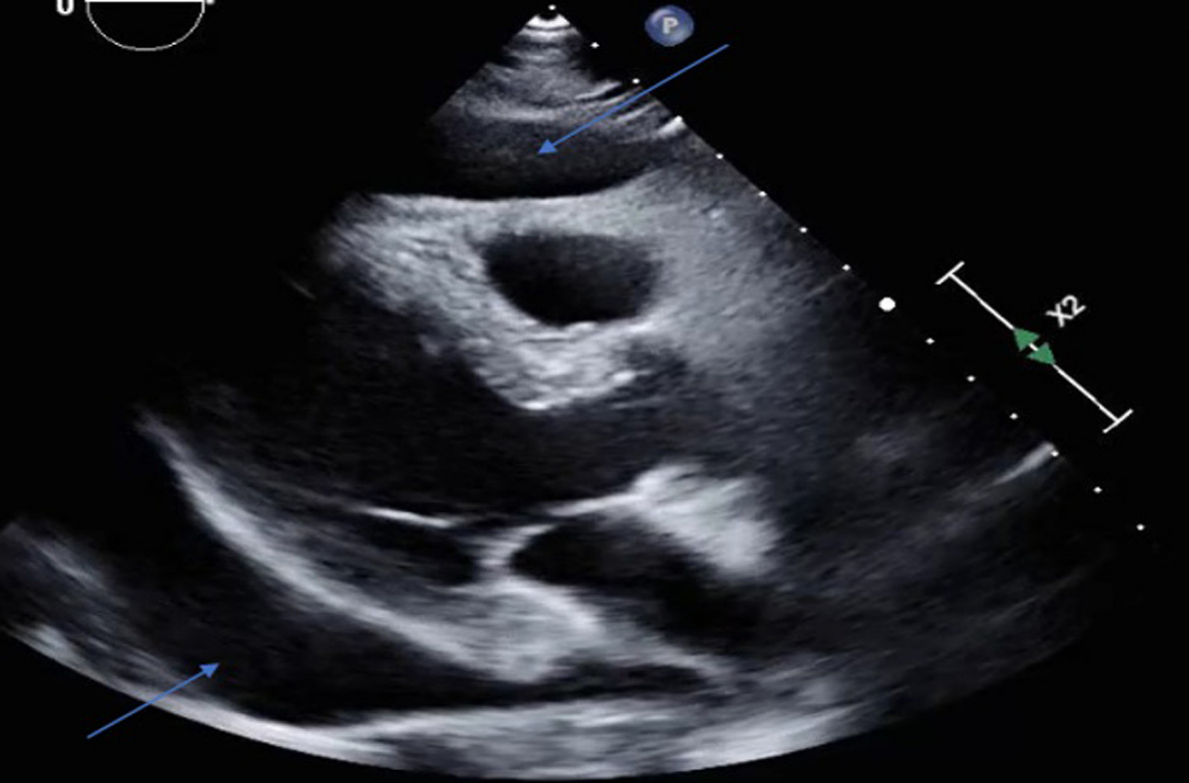
Transthoracic echocardiogram revealing large pericardial effusion.

**FIGURE 5:**
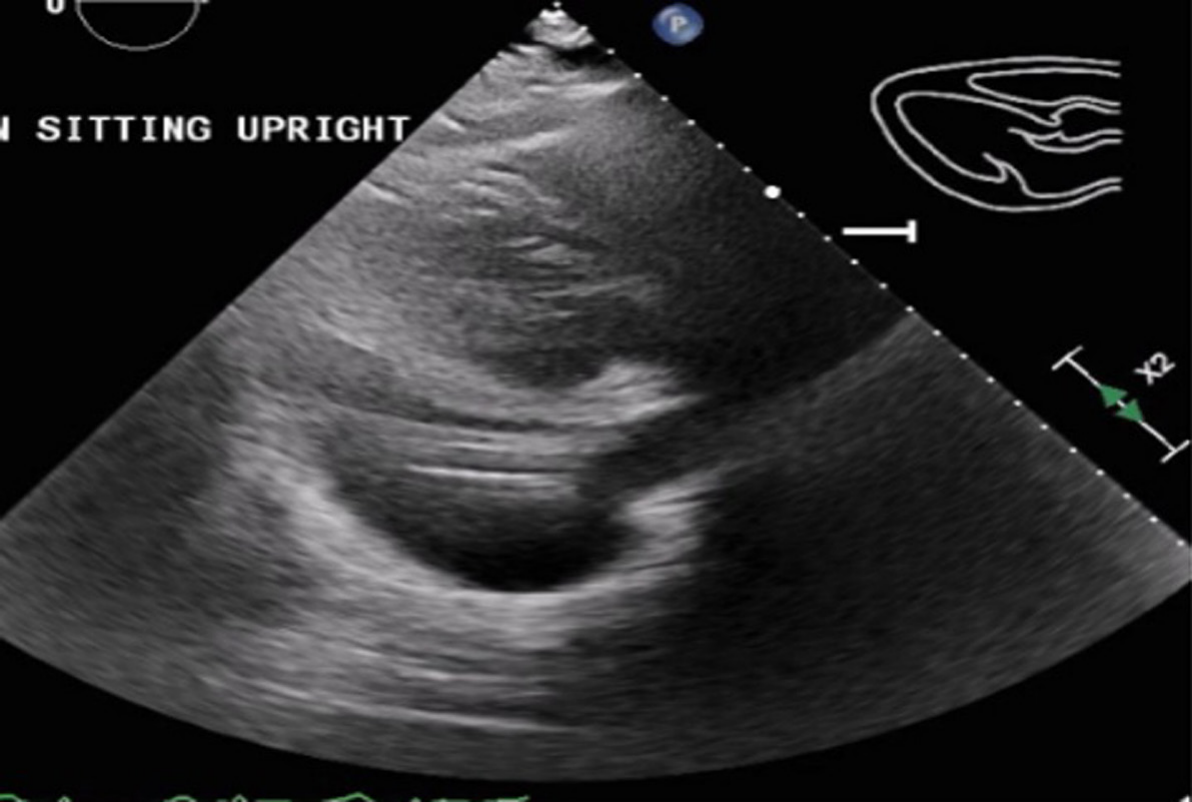
Echocardiogram revealing near complete resolution of pericardial effusion.

A transthoracic echocardiogram was performed and showed an ejection fraction of 65%, as well as a large pericardial effusion (Figure 4) measuring up to 3.1 cm in the apical views with evidence of diastolic right atrial and ventricular collapse, concerning for cardiac tamponade. Cardiothoracic surgery was consulted, and the patient subsequently underwent a subxiphoid approach pericardial window placement. Six hundred milliliters of bloody fluid was evacuated, fungal cultures, acid‐fast bacilli (AFB) culture with stains, cytology, and pericardial biopsy was obtained intraoperatively. A chest tube was placed postoperatively to monitor output. A repeat echocardiogram performed postoperatively showed the pericardial effusion had decreased significantly in size (Figure 5).

The patient's hospital course was further complicated by deep venous thromboses of the bilateral lower extremities for which an IVC filter was placed to prevent clot propagation. The chest tube that was placed postoperatively continued to drain minimal output and was subsequently removed. The patient was then discharged with close follow‐up with his oncologist the following week.

**FIGURE 6:**
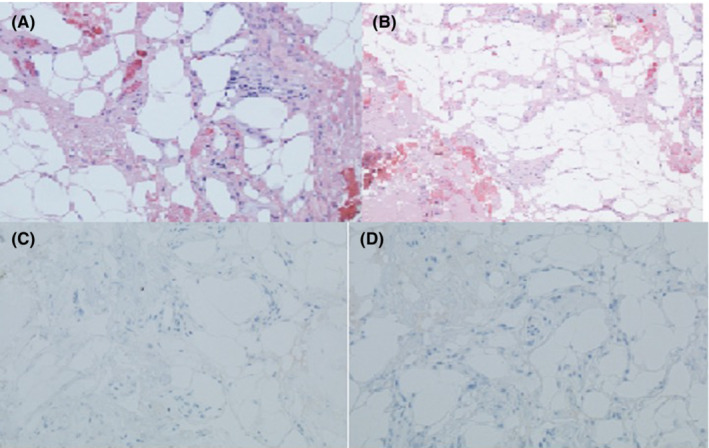
(A,B) H&E sections show fibroadipose tissue with minimal chronic inflammation. No tumor cells identified. (C,D) TTF‐1 and CK‐7 immunohistochemical stains are negative TTF‐1, thyroid transcription factor‐1, CK‐7, cytokeratin‐7.

Fungal cultures and AFB cultures with stains were negative after 4 weeks. The pericardial biopsy specimen was significant for fibrofatty tissue with nonspecific minimal chronic inflammation, no tumor cells identified, moderate WBCs seen and no organisms seen (Figure 6A,B). Thyroid transcription factor‐1 (TTF‐1) immunohistochemical stain was negative and cytokeratin 7 (CK7) immunohistochemical stain was negative as well, as shown in Figure 6C,D. Due to the concern of immunotherapy causing pericardial effusion and tamponade, the patient's pembrolizumab was held and the patient was maintained on pemetrexed and carboplatin.

## 3 DISCUSSION

The therapeutic value that immune checkpoint inhibitors have provided for patients with NSCLC since their approval in 2015 via the phase II/III Keynote‐010 trial has been unprecedented.^5^ Most notably overall survival and progression‐free survival have been found to be significantly superior in patients receiving immune checkpoint inhibitors when compared to the standard of care chemotherapy at that time. These outcomes continue to improve with greater understanding and application of these agents as time progresses. Shortly after initial approval, checkpoint inhibitors began receiving approval in other clinical applications of NSCLC. These indications included first‐line monotherapy in Stage 3 patients unable to undergo surgical resection, chemoradiation, or with metastatic NSCLC with PD‐L1 expression 1% or greater and no epidermal growth factor receptor (EGFR) or anaplastic lymphoma kinase (ALK) mutations.^5^ This approval paved the way for increased access to these medications with several more applications in clinical practice for different patient populations. However, with increased use of these agents came rare reports of immune‐related adverse events (irAEs) affecting multiple organ systems, sometimes resulting in life‐threatening consequences.^6^

The incidence of irAEs from pembrolizumab resulting in death was found to be 0.89 (95% CI, 00.49; 1.62, *I* ^2^, 0.00) in a meta‐analysis performed on 5090 patients.^6^ This emphasizes the importance of close monitoring while administering immune checkpoint inhibitors due to the rare but irrevocable complications that can occur. The irAEs associated with pembrolizumab that are reported in literature are: colitis, pneumonitis, hepatitis, various endocrinopathies,^6^ myocarditis, vasculitis, arrhythmias, and cardiac tamponade.^7^ The latter four are associated with the highest mortality. This case highlights pembrolizumab‐associated pericardial effusion causing cardiac tamponade and the pathophysiology, diagnostics, and treatment of this rare, but potentially fatal irAE.

Previous case reports of pembrolizumab‐induced pericardial effusion causing cardiac tamponade in the literature were first reported in 2016 with most cases requiring pericardiocentesis, pericardial window, withdrawal of pembrolizumab, and steroid treatment.^8^, ^9^, ^10^, ^11^ The median time from development of pericardial effusion to progression to cardiac tamponade after starting pembrolizumab has been reported to be about 3–4 months.^8^, ^10^ However, this time interval may be shorter or longer as sufficient data is lacking in the literature currently. Moreover, the pathophysiology behind the generation of the pericardial effusion itself is poorly understood. It has been hypothesized that the development of the effusion is due to disruption of the integrity of the pericardium due to blockade of the PD‐1:PD‐L1 complex, subsequently leading to increased T‐cell activity and inflammation.^12^ However, definitive claims cannot be made given the lack of controlled studies.

Pericardial effusion secondary to an immune checkpoint inhibitor such as pembrolizumab is also a diagnosis of exclusion. The diagnosis is nested primarily in a temporal relationship between the onset of symptoms and the initiation of the agent, the absence of another identifiable causes, and resolution once the agent has been discontinued.^12^ In our presented case, the patient's pericardial fluid showed moderate WBCs, but no growth of organisms on aerobic, anaerobic or fungal cultures while also being negative for AFB, effectively ruling out an infectious etiology. Given the lack of convincing evidence for other etiologies such as recent thoracic trauma and acute cardiac dysfunction, an effusion with tamponade development due to these causes was deemed less likely.

Additionally, a biopsy of the pericardium revealed only minimal chronic inflammation on histopathological assessment with no evidence of malignant cells, suggesting the process was acute and unrelated to involvement by the patient's malignancy. The pericardium also stained negative for TTF‐1 and CK7, which also further supports evidence against a carcinogenic etiology of the effusion.

Unfortunately, the exact pathophysiologic mechanism behind this patient's irAE remains unclear and no gold standard diagnostic test exists to aid the diagnosis, but further descriptions of pembrolizumab‐induced cardiac tamponade in the literature may eventually detail the pathologic process definitively in the future and lead to the development of such a modality.

Furthermore, only after considering the time frame, lack of evidence for more common etiologies of cardiac tamponade and the absence of pericardial effusion recurrence with withdrawal of pembrolizumab has this diagnosis of pembrolizumab‐induced cardiac tamponade been reached as a diagnosis of exclusion.

## 4 CONCLUSION

In conclusion, as immune checkpoint inhibitors increasingly become the mainstay of treatment for a growing number of cancers, so should an increased level of awareness with regard to possible life threatening irAEs. The occurrence of a large pericardial effusion is not a commonly noted adverse event associated with pembrolizumab and our report demonstrates that a rapid development can be diagnosed with close monitoring and triage to acute medical settings. However, more common additional diagnoses must be ruled out including infection, trauma, metastasis, and acute cardiac dysfunction before considering this diagnosis. Henceforth, imaging patients prior to the use of pembrolizumab could possibly be a preventive strategy to avoid this potentially lethal sequalae. However, more defined guidelines for significant effusions, time frame of development of the effusion and indications for definitive withdrawal of the checkpoint inhibitor must be further identified in a coordinated clinical trial setting. Therefore, in the interim, if clinical criteria alongside radiographic evidence correlates with a life threatening irAE such as cardiac tamponade, all measures should be taken to discontinue the offending agent and coordinate surgical intervention.

## AUTHOR CONTRIBUTIONS

**Dariusz Uczkowski:** Conceptualization; writing – original draft; writing – review and editing. **Hamza Ashraf:** Conceptualization; writing – original draft; writing – review and editing. **Arunabh Sekhri:** Formal analysis; investigation; supervision; writing – review and editing. **Arbaz Samad:** Data curation; resources; supervision; validation.

## FUNDING INFORMATION

No financial support was necessary for the preparation of this manuscript or acquiring data.

## CONFLICT OF INTEREST STATEMENT

All authors declare: no support from any organization for the submitted work; no financial relationships with any organizations that might have an interest in the submitted work in the previous 3 years; and no other relationships or activities that could appear to have influenced the submitted work.

## CONSENT

Written informed consent was obtained from the patient to publish this report in accordance with the journal's patient consent policy.

## Data Availability

The data that support the findings of this study are available on request from the corresponding author. The data are not publicly available due to privacy or ethical restrictions.
